# Prevalence and characteristics of ever regular use of non-combustible nicotine for 1 year or more: a population survey in England

**DOI:** 10.1186/s12954-021-00562-9

**Published:** 2021-11-17

**Authors:** Sharon Cox, Jamie Brown, Loren Kock, Lion Shahab

**Affiliations:** Department of Behavioural Science and Health, University College London, London, UK

**Keywords:** Nicotine, E-cigarettes, Tobacco cessation, Non-combustible nicotine, Smoking

## Abstract

**Introduction:**

Up-to-date monitoring of non-combustible nicotine products (e.g. e-cigarettes, nicotine replacement therapies (NRT), heated tobacco products (HTP); NNP) is important to assess their impact. To date, there is little evidence on the association between ever regular use (defined here as 1 year or more) of NNP and current smoking status.

**Aims/methods:**

The purpose of this study was to examine the prevalence, and sociodemographic, alcohol and smoking status correlates, of ever regular use of NNP in England in 2020. A cross-sectional survey of adults in England was conducted between February and June 2020.

**Results:**

A total of 8486 adults were surveyed; 94.9% (8055) were complete cases. The weighted prevalence of ever regular NNP use was 5.4% (n = 436; 95% CI 5.0–6.0), of which 82% (n = 360; 95% CI 78.7–85.8) was single and 18% (n = 79; 95% CI 14.8–22) multiple product use. Amongst ever regular NNP users, the prevalence of ever regular NRT, e-cigarette and HTP use was 64.7% (95% CI 60.1–69), 43.4% (95% CI 38.8–48) and 2.5% (95% CI 1.4–4.5), respectively. In adjusted analysis, ever regular NNP use was associated with smoking status, being significantly higher among current (22.3%; adjusted OR (aOR) 34.9, 95% CI 24.0–50.8) and ex-smokers (12.7%, aOR 19.8, 95% CI 11.1–14.4) than among never-smokers (0.6%). More advantaged occupational grade (aOR, 1.27 95% CI 1.02–1.57) and at least hazardous alcohol use (aOR, 1.38 95% CI 1.06–1.78) were associated with greater prevalence of ever regular NNP use.

**Conclusions:**

Ever regularly using NNP was highest among smokers and ex-smokers and rare among never-smokers. Among people who have ever regularly used NNP, NRT is the most popular.

## Introduction

In England, e-cigarettes and a range of licensed nicotine replacement therapies (NRT) are widely available and popular. There are high and moderate certainties evidence that NRT and e-cigarettes are effective for smoking cessation [3, 4, 14, 16, 20, 24]. More recently, nicotine salt “pod” e-cigarettes and heated tobacco products have also become available. The overall impact of the wide range of non-combustible nicotine products (NNP) on public health remains contested. Sources of contention include the extent to which the products are (1) used by people who would not otherwise have smoked, (2) used by smokers without quitting cigarettes, and (3) able to attenuate tobacco related inequalities. Monitoring the population who report past ever regular use of NNP, for extensive periods of time, can contribute to the evidence of their likely long-term impact and how ever regular use is associated with current smoking status. The purpose of this study is to examine the prevalence of, and sociodemographic and smoking-related characteristics, associated with ever regular use (defined here as 1 year or more) of NNP in England in 2020.

Patterns and duration of NNP use are associated with quit success. Regular and supported use of NRT is shown to yield higher quit rates than behavioural support alone [12, 20]. In England at the current time, e-cigarettes are the most popular quit aid, but use has been relatively stable since 2013 at approximately 5% of the adult population [26]. The most recent Cochrane review of the evidence suggests that with moderate certainty e-cigarettes are more effective than other types of nicotine replacement therapies and more effective than behavioural support alone [4]. However, although smokers report frequent experimentation with e-cigarettes, the proportion who use them regularly is much lower [11]. Also, regular daily use compared with irregular non-daily use by smokers is associated with a greater likelihood of smoking cessation [5, 11]. Further still, substantial health benefits are only gained when smokers switch to e-cigarettes completely [18], and the same is also true for other nicotine products. Therefore, in order for NNP to yield the best population health benefits use of these products must result in quitting. Furthermore, unintended consequences, such as never-smokers starting to use them, should be minimised. At the current time, in England there is some evidence that never-smokers use e-cigarettes and NRT [26], but the rates are extremely low, and whether this is short term or regular use over a longer term has not been clearly ascertained.

While there is a growing evidence base on the use of NRT and earlier generation e-cigarettes [26, 28], there is a paucity of evidence on relatively newer pod nicotine-salt e-cigarette devices (e.g. “JUUL”). The relatively low cost of e-cigarette use compared with tobacco and simplicity of JUUL may be appealing to a proportion of smokers who report finding tradition tank like e-cigarettes cumbersome [21]. However, some have raised concern that the ease of use of JUUL that is appealing to people who smoke could lead to more recreational use of nicotine amongst never-smokers and young (nicotine naïve) people [1]. These concerns have been set against a rise in popularity of JUUL in the USA. It is therefore useful to monitor use of this product as a separate product of interest in England, so to monitor the uptake and use within a different regulatory context.

At the current time, there is less published evidence on heated tobacco products (HTP), especially in relation to prevalence and correlates of ever regular use. It is important to monitor use of these products, because some people who smoke may find HTP especially appealing because they offer a similar sensory and psychological experience to smoking [22], and e-cigarettes do not satisfy many people who try them [23]. However, the high start-up cost of HTP may be a barrier to initiation and regular use for those from lower-income groups. Monthly data from England show very low current use of both JUUL and HTP compared with “typical” e-cigarettes and NRT [26], and to date, there are no data on longer-term use of these products.

Monitoring *regular* ever use across NNP by sociodemographic factors and current smoking status can help to understand which groups are most likely to benefit from the wide array of products, shape public health messages and monitor unintended consequences. Early evidence showed that e-cigarettes were most likely to be used those from more advantaged social grades [25], but a more recent analysis by Kock et al. [10] showed that in England e-cigarette use among > 1-year ex-smokers increased among all occupational social grades from 2014 to 2017 and use was highest among those within more disadvantaged occupational social grades [10]. Age is also an important factor to explore, as older adults are still more likely to choose NRT over all other products despite the growing popularity of e-cigarettes [26]. Other substance use behaviours which are highly correlated such as alcohol are also important to explore, as substance behaviours which co-exist play a role in the use of one substance in the presence of another [15]. However, co-use in this context may be more reflective of the need to use nicotine than tobacco, as evidence highlights recent ex-smokers who used e-cigarettes or NRT to quit smoking consume more alcohol than those who quit unaided [6].

Using monthly cross-sectional data from the Smoking Toolkit Study (STS) collected between February 2020 and June 2020, this present study examined the prevalence and characteristics of ever regular use, defined here as 1 year or more, of non-combustible nicotine products in England. Specifically, the following research questions were examined:

1. What is the prevalence of ever regular use of i) non-combustible nicotine products (NNP), ii) NRT, iii) E-cigarettes iv) JUUL and v) heated-tobacco products?
2. Is ever regular use of each outcome associated with socio-demographic characteristics, smoking status and alcohol use?

## Methods

### Design and participants

Data were drawn from the ongoing STS, a monthly repeated cross-sectional survey of a representative sample of adults (≥ 16 years) in England [2]. Each month, a form of random location in combination with quota sampling is used to select a new sample of approximately 1700 adults aged 16 years and older. Further details on the design of the STS, including sampling and weighting technique, can be found elsewhere [2]. Standard protocol means data are usually collected monthly through face-to-face computer-assisted interviews. However, due to the COVID-19 pandemic and rules on social distancing, from April 2020 data were collected via telephone among adults aged 18 and over only. The telephone-based data collection relied on the same combination of random location and quota sampling, and diagnostic analyses have demonstrated comparable results from before and after the lockdown, despite the modified data collection method [7]. For the present study, we used aggregated data from the time period that the question relating to ever regular NNP use was introduced into the survey.

### Measures

#### Outcome variable

To assess ever regular NNP use, all respondents were asked “Can I check, have you ever regularly used any of the following for a year or more?”, responses included 1. nicotine gum, 2. nicotine lozenge, 3. nicotine patch, 4. nicotine inhaler\inhalator, 5. nicotine mouth spray, 6. electronic cigarette, 7. heat-not-burn cigarette (e.g. iQOS, heatsticks), 8. JUUL. Responses for each item were coded into a binary yes/no. Product use is presented as a total of all non-combustible product use. Categories was computed based on the following, NRT (derived by responses 1–5), e-cigarette (6), heated tobacco products (7), and JUUL (8). Multiple product use was calculated based on yes responses to two or more products. The addition of nicotine pouches was not included in this study as entry into the STS was after the study data collection period.

Due to a filtering error, 11 people who reported daily e-cigarette use were not asked the above question. In order to establish ever regular NNP use, the following question was used as an inclusion check, “Can I check, are you using any of the following either to help you stop smoking, to help you cut down or for any other reason at all?” and for those who said yes, a further question “How long have you been using this nicotine replacement product or these products for?”, 1. Less than 1 week, 2. One to 6 weeks, 3. More than 6 weeks and up to 12 weeks, 4. More than 12 weeks and up to 26 weeks, 5. More than 26 weeks and up to 52 weeks, 6. More than 52 weeks. Seven participants reported current e-cigarette use for a period of greater than 12 weeks and were not asked about ever regular e-cigarette use, and these participants were recoded as ever regular e-cigarette users.

#### Explanatory variables

*Smoking status* Those who reported currently smoking cigarettes or tobacco of another type were considered to be a smoker. All of those who reported having stopped smoking within the last year or before were considered ex-smokers. All others were considered never-smokers.

*Alcohol use* To test the association between hazardous alcohol use across product categories, scores on the Alcohol Use Disorder Identification Test (AUDIT) were dichotomised into those scoring 8 and above vs less (hazardous alcohol use vs not).

*Socio-demographic characteristics* In the present study, we examined self-reported gender was categorised as women or other, and actual age is categorised as 16–24, 25–34, 35–44, 45–54, 55–64 and ≥ 65 years.

Occupation-based social grade (C2DE includes manual routine, semi-routine, lower supervisory and long-term unemployed; ABC1 includes managerial, professional and upper supervisory occupations) and children in the household (yes/no).

*Analyses* The protocol for this study was preregistered on the Open Science Framework (https://osf.io/fmypr/). Analyses were conducted using SPSS v25 on complete cases. Data were weighted to match the English population profile on age, social grade, region, tenure, ethnicity and working status within sex and were weighted for all analyses. The dimensions are derived monthly from a combination of the English 2011 census, Office for National Statistics mid-year estimates, and an annual random probability survey conducted for the National Readership Survey. For the first research question, we report the prevalence (and 95% confidence interval [CI]) ever regular NNP use; this includes use of all NRT products, e-cigarettes and all heated tobacco products. Logistic regression estimated the association of ever regular use of each outcome with smoking status, at least hazardous drinking and sociodemographic characteristics, with and without mutual adjustment. Goodness-of-fit tests indicated the full model statistically significantly predicted the dependent variable better than the intercept-only model alone (Likelihood ratio < 0.001). Independence of observations and multicollinearity were evaluated with simple correlations among the independent variables.

## Results

Across five waves from February 2020 to June 2020 a total of 8486 adults in England were surveyed and 94.9% (8055) were complete cases. The weighted prevalence of ever regular non-combustible nicotine use was 5.4% (n = 436; 95% CI 4.9–5.9) of which 82.6% (n = 360; 95% CI 78.70–85.8) was single and 18.1% (n = 79; 95% CI 14.8–22) multiple product use. Within those reporting ever regular NNP use the prevalence of ever regular NRT, e-cigarette and heated tobacco product use was 64.7% (n = 282; 95% CI 60.1–69), 43.4% (n = 189; 95% CI 38.8–48) and 2.5% (n = 11; 95% CI 1.37–4.54), respectively. Ever regular use of JUUL was not reported (0%).

Ever regular use of any NNP use was highest for current smokers (22.3%, n = 205; 95% CI 19.8–25.1), followed by ex-smokers (12.7%, n = 198; 95% CI 11.1–14.4) with negligible use by never-smokers (0.6%, n = 33; 95% CI 0.5–0.9). Ever regular use of NRT or e-cigarettes was each more prevalent in current smokers (11%, n = 124; 95% CI 9.3–13 and 9.3%, n = 104; 95% CI 7.7–11.1, respectively), followed by ex-smokers (8.2%, n = 145; 95% CI 7.1–9.6 and 3.6%, n = 63; 95% CI 2.8–4.6, respectively), with very low use in never-smokers (0.2%, n = 11; 95% CI 0.1–0.4 and 0.4%, n = 21; 95% CI 0.3–0.6, respectively). Ever regular use of HTP was rare across current smokers (0.4%, n = 4, 95% CI 0.1–1), ex-smokers (0.2%, n = 4, 95% CI 0.1–0.7) and never-smokers (0.1%, n = 3, 95% CI 0.1–0.7).

Table 1 presents sample characteristics of those reporting use and bivariate and multivariable associations between sociodemographic characteristics, hazardous alcohol drinking and smoking status across product categories.

**Table 1:** Sample descriptive characteristics and factors associated with ever past regular combined non-combustible nicotine product use (NRT, e-cigarettes and heated tobacco products combined) and NRT products, e-cigarettes

	Whole sample (N = 8486)*	Non-combustible nicotine products combined (NNP: N = 436)			Nicotine replacement therapies (NRT: N = 282)			E-cigarettes (N = 189)		
	% (n)	%	OR (95% CI) *p* value	Adj OR (95% CI) p value	%	OR (95% CI) * p* value	Adj OR (95% CI) * p* value	%	OR (95% CI) * p* value	Adj OR (95% CI) * p* value
*Age*										
16–24	11.2 (902)	9.3	1	1	4.6	1	1	15.3	1	1
25–34	15.4 (1240)	18.9	1.47 (1.01–2.17) 0.05	1.33 (0.87–2.04) 0.19	12.4	1.92 (1.01–3.65) 0.05	1.66 (0.83–3.38) 0.15	29.1	1.36 (0.86–2.15) 0.19	1.22 (0.79–2.03) 0.46
35–44	14.7 (1185)	17.5	1.44 (0.97–2.12) 0.68	1.47 (0.94–2.30) 0.09	18.4	**3.07 (1.66–5.66) < 0.001**	**2.92 (1.49–5.71) 0.002**	18.5	0.88 (0.54–1.45) 0.62	0.92 (0.53–1.61) 0.77
45–54	16.0 (1286)	17.5	1.31 (0.89–1.94) 0.17	1.37 (0.89–2.13) 0.15	20.6	**3.16 (1.72–5.80) < 0.001**	**3.37 (1.70–6.28) < 0.001**	13.2	0.60 (0.35–1.02) 0.59	0.62 (0.34–1.11) 0.11
55–64	14.3 (1149)	16.6	1.39 (0.94–2.06) 0.10	1.36 (0.88–2.13) 0.17	18.8	**3.23 (1.75–5.95) < 0.001**	**3.05 (1.57–5.90) < 0.01**	12.2	0.61 (0.35–1.05) 0.74	0.64 (0.34–1.14) 0.12
65+	22.8 (1838)	19.8	1.04 (0.71–1.52) 0.85	0.99 (0.64–1.56) 1.00	24.8	**2.70 (1.49–4.91) 0.001**	**2.49 (1.29–4.83) 0.007**	11.1	**0.34 (0.19–0.60) < 0.001**	**0.37 (0.20–0.71) 0.002**
*Sex*										
Male	46.2 (3720)	49	1	1	45.7	1	1	58.2	1	1
Female	48.4 (3896)	51	0.92 (0.76–1.12) 0.40	1.12 (0.90–1.37) 0.32	54.3	1.15 (0.91–1.46) 0.25	1.44 (1.10–1.86) **0.01**	41.8	0.69 ( 0.51–0.92) 0.69	0.78 (0.57–1.07) 0.12
*Social grade*										
C2DE	44.5 (3532)	48.3	1	1	48.8	1	1	47.1	1	1
AB/C1	55.5 (4408)	51.7	0.85 (0.70–1.03) 0.11	**1.27 (1.02–1.57) 0.03**	51.2	0.84 (0.66–1.06) 0.14	1.20 (9.22–1.55) 0.18	52.9	0.90 (0.67–1.20) 0.46	**1.50 (1.06 – 2.01) 0.02**
*Children in the home*										
No	66.8 (5377)	70.6	1.00	1.00	71.6	1.00	1.00	66.3	1.00	1.00
Yes	27.8 (2239)	29.4	0.99 (0.81–1.23) 0.98	0.91 (0.71–1.20) 0.51	28.4	1.06 (0.81–1.38) 0.68	0.91 (0.65–1.26) 0.91	33.7	0.82 (0.60–1.11) 0.20	0.99 (0.69–1.42) 0.95
*Smoking status*										
Never	63.7 (818)	7.6	1	1	3.9	1	1	11.2	1	1
Ex-smoker	19.4 (1562)	45.4	**19.81 (13.63–28.80) < 0.001**	**19.84 (13.50–29.15) < 0.001**	51.8	**42.45 (22.89–78.73) < 0.001**	**37.30 (20.02–69.49) < 0.001**	33.5	**9.02 (5.49–14.82) < 0.001**	**11.25 (6.71–18.86) < 0.001**
Current	11.4 (918)	47.7	**34.88 (23.96–50.80) < 0.001**	**35.34 (23.96–52.13) < 0.001**	44.3	**58.27 (31.26–108.61) < 0.001**	**63.68 (33.89–119.65) < 0.001**	55.3	**24.85 (15.48–39.90) < 0.001**	**23.53 (14.31–38.69) < 0.001**
*Hazardous drinking*^*+*^										
No	82.5 (6541)	77.5	1	1	78.1	1	1	78.6	1	1
Yes	12.2 (968)	22.5	**0.51 (0.40–0.65) < 0.001**	**1.38 (1.06–1.78) 0.02**	21.9	**0.54 (0.40–0.73) < 0.001**	**1.42 (1.04–1.95) 0.03**	21.4	**0.56 (0.39–0.81) 0.002**	1.08 (0.74–1.58) 0.69

Figures in bold represent statistical significance *p* < 0.05

*Weighted sample so figures do not always precisely match the whole sample

^**+**^Hazardous drinking as identified by scores between 8 and 15 on the AUDIT

In the adjusted model, being a current or ex-smoker, being of social grade (ABC1) compared with (C2DE) and at least hazardous alcohol use were significantly associated with greater prevalence of ever regular NNP use. This pattern of associations were similar for each product type with a few exceptions: the association with social grade was only observed with ever regular e-cigarette but not NRT use, whereas the opposite was true for the association with at least hazardous alcohol use (Table 1). In addition, there were also some product-specific associations: older age was associated with greater odds of ever regular NRT use, but lower odds of ever regular e-cigarette use and female sex was also independently associated with greater ever regular NRT use (Table 1). HTP ever regular use was low (n = 11), and therefore, individual associations were not calculated.

## Discussion

In this representative sample of adults in England, ever regular use of NNP was almost entirely reported by current or ex-smokers, and use of any type of nicotine product by non-smokers was minimal. Ever regular use of NRT was most prevalent, followed by e-cigarettes and then heated tobacco products. Ever regular use of heated tobacco products was low, and nobody in this survey reported ever regular use of the e-cigarette, JUUL.

These results extend the current literature in several ways. Firstly, it is important to monitor uptake and use of NNP across different groups including non-smokers; the results here corroborate other surveys and reviews showing that long-term or extended use of NNP is almost exclusively linked to past or current smoking [14]. This finding is important as there have been concerns that, especially e-cigarettes, may increase the appeal of nicotine use in never-smokers, but there is little evidence of ever regular use reported here. Second, ever regular NRT use was more prevalent than ever regular e-cigarette use. This may in part be explained by the much greater time period over which NRT has been available to be regularly used by a much larger cohort of people who were at the time it was available current smokers.

As has been found in other UK surveys of heated tobacco products, use of these products is very low in comparison with other NNP [13]. There are several plausible explanations for this relating to accessibility of the product (limited retail outlets) and also the price of the product. As heated tobacco products are subject to higher duty, use of the product offers little price advantage, and as has been documented elsewhere, price is a key driver for product use and motivation to quit smoking [8, 9, 21]. Lastly, in relation to prevalence, nobody from just over 8000 people reported ever regular use of the e-cigarette JUUL. Concerns have been raised that the “gadgetry” and ease of use of JUUL could appeal to never-smokers, especially young never-smokers [1], but there is no evidence of regular use reported here, even among smokers and ex-smokers. It may be that this particular brand is not as appealing in competition with other well-established types of e-cigarette brands and other types of devices, e.g. tanks or other pods, which were widely used and available on the market before the launch of JUUL, and the UK has enacted stricter regulatory control around marketing compared to the USA. Differences in trends of use between the UK and the USA may also be attributed to the difference in nicotine strength availability (USA 59 mg/mL and UK up to 20 mg/mL). However, monitoring trends in pod more broadly is useful as a number of smokers have reported not liking traditional tank devices or finding them difficult to use [21].

Use of multiple NNP was lower than single use. The design of this study means we are not able to infer cessation, but if participants are using these products to quit smoking, then use of a single product is potentially sub-optimal, as a recent Cochrane review of the evidence shows that use of two nicotine replacement products, fast acting combined with slow release, results in higher quit rates than single use [12]. Furthermore, a recently published randomised control trial by Walker et al. [24] showed that use of an e-cigarette with nicotine e-liquid alongside a transdermal nicotine patch leads to modest but higher CO verified quit rates at 6-months compared with an e-cigarette with nicotine-free e-liquid or a patch alone (7%, 4% and 2%, respectively). We are also not able to assess how and in which combination the products were used. This is important future work, as the products available on the English market continue to grow, establishing which combination of products and how they are used may be most effective compared to single use is an important focus for future trials.

In relation to the sociodemographic associations, overall the results are in line with previously published work [8, 9]. Ever regular use of NNP was more prevalent in those from occupational grades ABC1 and use of alcohol to at least hazardous levels. Similarly, NRT was associated with female sex, older compared with younger 16–24-year-old adults and at least hazardous drinking. E-cigarettes were most commonly ever regularly used by younger adults, and there is decline in use by age; again, this has been reported elsewhere [8, 9].

Those respondents from occupational social grades C2DE reported less ever regular e-cigarette use than those from more advantaged occupational grades (ABC1), but there was no social gradient effect in ever regular use of NRT. In England at the current time, smoking prevalence rates are twice as high in the least advantaged occupational grades compared with those in more advantaged professions [27]. Recent trial evidence has shown that e-cigarettes offered within the English stop smoking services provide the best chances of quitting tobacco and remaining abstinent at 1 year [3], although the impact of e-cigarettes on reducing the social gradient of tobacco use is yet to be established. Nonetheless, regular use of e-cigarettes is associated with complete switching [5], so how best to encourage and support those people smoking from groups with a high smoking prevalence to both initiate e-cigarette use and to use their devices more regularly to prevent relapse to smoking and reduce concurrent tobacco use requires more consideration.

There is conflicting evidence on the use NNP by sex [19], but here females report higher use of NRT. Evidence from across health sciences show men often require more encouragement to engage in formal health promoting interventions [17]. We also found an inverse association with age, with ever regular use of NRT more likely by adults over 35 years and of e-cigarettes by adults below this age. These age effects are not surprising given NRT products have been widely available for a longer period of time. However, speculatively e-cigarettes may be more appealing to younger users than NRT and this could also reflect a diffusion of technology, e.g. older smokers adopt e-cigarette use at a reduced pace compared with younger smokers. Lastly, in line with our recent work, a higher level of self-reported alcohol use was associated with NNP use and NRT [6]. This may indicate that users who are more nicotine dependent are more likely to drink alcohol. This may signal that for those smokers who drink alcohol regularly additional support is required to stop smoking and specifically tailored support to help them in situations when they are consuming alcohol and or they cannot smoke (i.e. for temporary abstinence).

This study offers a useful understanding of which groups are using NNP and how ever regular use of 1 year or more is associated with current smoking status; however, there are some limitations. No causal or temporal associations with cessation can be made as this analysis is cross-sectional only and we have not reported here why people were using these products, but this does lead to important questions for future research. We inferred a small sample (n = 7), of people currently using e-cigarettes were regular users—and therefore ever regular users—if they reported using products for more than 12 weeks (beyond standard length of time products are used for smoking cessation). Other limitations include potential recall bias, that is self-reporting is not always accurate, and regular use may be considered differently across individuals, future research should try to capture more granular patterns of use although this may be hard over more sustained periods of time. Our data were collected during the early part of the COVID-19 pandemic in England; this may have impacted the sample or nature of information provided as respondents were interviewed by telephone instead of face-to-face.

Future studies should try to gain a more detailed pattern of regular use, especially key differences in the trajectory of ever regular use between current smokers, ex-smokers and dual users, that is, understanding how usage patterns, frequency and duration, are associated with helping people quit smoking and remain quit. Furthermore, a high proportion of smokers report regularly using NRT and e-cigarettes, what is not known is whether these individuals reached a period of abstinence and then relapsed or whether they relapsed back to smoking. We observed clear effects of ever regular product use by age, and future research could focus on the distinct needs of older versus younger smokers, including whether e-cigarettes are not appealing to older smokers or whether lower uptake reflects product diffusion, or both. Lastly, there has been an increase in the number of people attempting to quit smoking in England during the COVID-19 pandemic [7], but also increased stress and uncertainty, so while people may be attempting to use NNP to quit smoking, how this transfers into longer-term use over this unique period will require further monitoring.

## Conclusion

To conclude, regular NNP use of 1 year or more is associated with current smoking or ex-smoking and we report little use amongst never-smokers, this is an important finding as with the rise of NNP there has been concerns raised regarding use by never-smokers. If never-smokers are using these products, then it does not appear to be habit forming. It therefore remains the case that non-combustible nicotine research should focus on how best to help people who smoke to quit. However, e-cigarette use was also independently associated with more advantaged occupational grades and given the large burden of smoking on health inequalities how best to support smokers from less advantaged positions to switch to safer forms of nicotine and stay switched remains an important focus for future work.

## Data Availability

The protocol for this study can be found at Open Science Framework (https://osf.io/fmypr/). The data output will be available at this page after publication.

## Abbreviations

NNP: Non-combustible nicotine product/s
NRT: Nicotine replacement therapies
E-cigarette: Electronic cigarette
